# Implementation of a proactive referral tool for child healthcare professionals to encourage and facilitate parental smoking cessation in the Netherlands: a mixed-methods study

**DOI:** 10.1186/s12913-021-06969-1

**Published:** 2021-09-16

**Authors:** Tessa Scheffers-van Schayck, Bethany Hipple Walters, Roy Otten, Marloes Kleinjan

**Affiliations:** 1 Trimbos Institute, Netherlands Institute of Mental Health and Addiction, P.O. Box 735, 3500 AS Utrecht, the Netherlands; 2 Department of Interdisciplinary Social Science, Utrecht University, P.O. Box 80125, 3584 CS Utrecht, the Netherlands; 3 Division of General Academic Pediatrics, Massachusetts General Hospital for Children, Boston, USA; 4 Clinical Developmental Psychology, Radboud University, P.O. Box 9104, 6500 HE Nijmegen, The Netherlands

**Keywords:** Smoking cessation, Parents, Proactive referral tool, Healthcare professionals, Child healthcare settings, Second-hand smoke, Implementation study, Mixed-methods

## Abstract

**Background:**

Recently, the parent-tailored telephone based smoking cessation counseling program ‘Smoke-free Parents’ was shown to be effective in helping parents to quit smoking. To implement this program in child healthcare settings in the Netherlands, the research team developed a proactive referral tool to refer parents to Smoke-free Parents. The aim of the present implementation study was to explore the facilitators, barriers, and suggestions for improvement in the implementation of this referral tool.

**Methods:**

Child healthcare professionals (*N* = 68) were recruited via multiple strategies (e.g., social media, mailings, and word of mouth among healthcare professionals) and invited to complete two online (quantitative and qualitative) questionnaires and to participate in a telephone semi-structured qualitative interview between April 2017 and February 2019. In total, 65 child healthcare professionals were included in the analyses. After inductive coding, thematic analyses were performed on the qualitative data. Descriptive analyses were performed on the quantitative data.

**Results:**

The data from both questionnaires and the telephone interview revealed that the majority of the child healthcare professionals (92.3 % female; average years of working as a healthcare professional: 23.0) found the Smoke-free Parents referral tool accessible and convenient to use. Yet there were several barriers that limited their use of the tool. The data revealed that one of the main barriers that healthcare professionals experienced was parental resistance to smoking cessation assistance. In addition, healthcare professionals noted that they experienced tension when motivating parents to quit smoking, as they were not the parent’s, but the child’s healthcare provider. Additionally, healthcare professionals reported being concerned about the lack of information about the costs of Smoke-free Parents, which limited professionals referring parents to the service.

**Conclusions:**

Although healthcare professionals reported rather positive experiences with the Smoke-free Parents referral tool, the use of the tool was limited due to barriers. To increase the impact of the Smoke-free Parents telephone-based smoking cessation counseling program via child healthcare settings, it is important to overcome these barriers. Suggestions for improvement in the implementation of the referral tool in child healthcare settings are discussed.

## Background

### Recruiting parents for parent-tailored smoking cessation interventions via child healthcare settings

Worldwide, more than half a billion children are exposed to secondhand smoke (SHS) at home [1]. Children are primarily exposed to SHS by parental smoking [2]. Children who are exposed to SHS are more likely to develop respiratory illnesses, including asthma and bronchitis, and to initiate smoking in the future [3–5]. To decrease the adverse health consequences for parents (e.g., respiratory and cardiovascular diseases, and cancer [6]) and their children, and to reduce the chance that children start smoking, it is paramount that smoking parents receive evidence-based smoking cessation interventions that help them to quit smoking.

Healthcare professionals play an essential role in identifying smokers, motivating them to quit smoking, and offering them evidence-based smoking cessation treatment [7]. National clinical practice guidelines have been developed in several countries to help healthcare professionals to address smoking cessation and provide treatment [7–9]. A frequently used approach is Ask-Advise-Refer (AAR), whereby healthcare professionals *ask* patients about their tobacco use, *advise* smokers to quit, and reactively *refer* smokers who are interested in quitting to evidence-based cessation interventions (e.g., a quitline) [10–12]. Reactive referral means that smokers receive the contact information of smoking cessation services and are encouraged to initiate contact themselves [10, 13]. In contrast to reactive referral, healthcare professionals can proactively refer parents to evidence-based smoking cessation interventions, i.e. smokers are directly contacted by the smoking cessation services. Research suggests that proactive recruitment strategies could increase the inclusion of smokers into smoking cessation interventions compared to reactive recruitment strategies [14, 15].

About ten years ago, a proactive parent-tailored telephone smoking cessation counseling program was developed in the Netherlands [16]. This counseling program included up to seven telephone calls (initiated by certified smoking cessation counselors) during a period of three months and a parent-tailored brochure on smoking cessation [16]. A randomized controlled trial (RCT) showed the effectiveness of this telephone counseling program at three and twelve months follow-up [17]. Based on the promising results of this efficacy trial, the research team developed and conducted a follow-up trial using an effectiveness-implementation hybrid design [18–20]. This follow-up trial had two aims. The first aim was to examine the effectiveness of the Dutch telephone counseling program (now called “Smoke-free Parents” (SFP)) in a more real-world setting (i.e., an effectiveness trial). More information on the rationale behind and the results of this effectiveness trial can be found elsewhere [21, 22]. The second aim was to examine in an implementation study how SFP could be implemented in – among others – healthcare settings [21, 22]. Child healthcare settings were selected as proactive recruitment approach, because it was assumed that parents are receptive to smoking cessation interventions in these settings and these settings could serve as a “teachable moment” [23–25]. In the Netherlands, child healthcare professionals are in principle child’s healthcare providers and not the healthcare providers of adults (except for the care that is provided before birth). Children between 0 and 17 years receive different types of child healthcare in the Netherlands (see Table 1). For example, medical child healthcare professionals (e.g., general practitioners and pediatricians) provide medical care to children. Specialized child healthcare professionals (e.g., child healthcare clinicians and nurses) provide preventive healthcare that includes – among others – monitoring children’s development, providing vaccinations to children, enabling early identification of any problems, and referring children to specialist care if needed [26]. For this type of healthcare, parents are encouraged to visit youth healthcare centers 15 times in the first four years of their child’s life. In addition, when children are in primary and secondary school they have regular check-ups at school. Because around 95 % of Dutch parents with children between the ages of 0 and 4 years reported to have visited youth healthcare centers [27], these centers could be a viable implementation setting for SFP.

**Table 1:** An overview of different types of child healthcare in the Netherlands

Age of child	Care providers	Care location	Type of care provided
Before birth	Midwives, gynecologists, and obstetrician	Midwifery practices Hospitals	Women receive prenatal care during their pregnancy
0–2 weeks old	Maternity nurses	Care at home	Women and infants receive postnatal care for a couple of hours during the first eight days postpartum
0–17 years old	Specialized child healthcare professionals (i.e., child healthcare clinicians and nurses)	Youth healthcare centers and schools	Children’s physical/cognitive/behavioral development is being monitored and children receive multiple vaccinations throughout the years
0–17 years old	Medical child healthcare professionals (i.e., general practitioners, pediatricians, and other child healthcare clinicians and nurses)	General practices, hospitals, and medical clinics	Children receive medical care if needed

### The development of an implementation strategy for SFP in child healthcare settings

To support implementation in child healthcare settings, the research team developed a comprehensive implementation support strategy. The research team first organized and hosted several expert meetings with ten representatives of Dutch child healthcare professionals preliminary to the follow-up trial. The aim of these meetings was to develop an implementation strategy to implement SFP in child healthcare settings. During these meetings, these professionals stated that they needed a system that enables them to proactively refer smoking parents to SFP. Based on this information and on the evidence of proactive recruitment [14, 15], the research team developed a proactive referral tool in collaboration with the healthcare professionals as an implementation strategy.

The referral tool consisted of two main steps. First, healthcare professionals could use the tool to refer parents to SFP (online, by fax or by phone) if parents were interested in quitting smoking and willing to receive evidence-based treatment. Second, a smoking cessation counselor from SineFuma (one of the Dutch certified quit lines) approached parents who were referred to SFP within one week. During this brief (5–10 min), free, informative, and proactive phone call, counselors provided more information about SFP (e.g., the costs of the program (between € 300 and € 370) that parents might need to pay if their health insurance did not reimburse the costs). Parents could withdraw or confirm their registration for SFP during this phone call (please see Scheffers-van Schayck et al. [2021] [28] for more information and results on the implementation of the second step of the SFP referral tool).

During the expert meetings, the representatives also mentioned to be in need of a toolkit that could support healthcare professionals with using the referral tool. Therefore, the research team developed a free toolkit as an implementation strategy in collaboration with the representatives. The toolkit included: (1) a paper-based information card (size A5) for healthcare professionals that provided information about the referral tool; (2) a small paper-based card (size A6) that healthcare professionals could give to parents to inform them about SFP and the risks of children’s exposure to SHS; and (3) a poster of SFP. Healthcare professionals who signed up for the referral tool (and the implementation study, see “Methods” for more information) received the toolkit by mail and were asked to disseminate the toolkit among their co-workers.

The final implementation strategy of the research team was to contact healthcare professionals by phone when they had signed up for the referral tool (and the implementation study). The aim of this phone call was to inform healthcare professionals about the SFP referral tool, toolkit, and the implementation study. Because the referral tool was developed with the aim to be convenient in its use as much as possible and the instructions were provided in the toolkit, the healthcare professionals received no additional face-to-face training. However, if healthcare professionals had some questions about the referral tool and/or toolkit, they approached the research team by phone and e-mail.

### The present implementation study

Currently, it is unknown what the experiences were of healthcare professionals who used the SFP referral tool. Therefore, as part of the overall follow-up trial, the present implementation study aimed to explore the facilitators, barriers, and suggestions for improvement in the implementation of the proactive SFP referral tool in child healthcare settings. These facilitators and barriers could concern characteristics of the referral tool itself (e.g., the materials of the referral tool), but also factors that affect the use of the referral tool (e.g., the barriers that healthcare professionals experience in discussing smoking cessation with parents). The data collected through interviews and questionnaires yield essential insights into the experiences of healthcare professionals and provide important directions for further development and widespread implementation of the tool in child healthcare settings.

## Methods

### Design and ethics

The present implementation study is part of a large trial that uses an effectiveness-implementation hybrid design [18–20]. An effectiveness-implementation hybrid design is one that takes a dual focus a priori in assessing clinical effectiveness and implementation of, in this case the Dutch proactive telephone smoking cessation counseling SFP tailored to parents of children aged 0–18 years. The clinical effectiveness and program uptake among parents are published elsewhere [22, 28]. The large trial was registered in the Netherlands Trial Register (NTR6092; registration date: 19/09/2016). Further information on the complete study design can be found elsewhere [21, 22]. The ethics committee of the Trimbos Institute approved this study’s protocol (201607_52-1606).

### Participants and recruitment

 In the present study, two groups of child healthcare professionals participated: medical child healthcare professionals and specialized child healthcare professionals. Healthcare professionals involved in antenatal care (e.g., fertility doctors) did not participate in the present study because the smoking cessation program was aimed at current parents and not at for example people with a child wish. During the study, three midwives who heard about our study indicated to be in need of a tool that enabled them to refer pregnant women to smoking cessation support. Because the quit line that delivered the Smoke-free Parents program also offered smoking cessation support tailored to pregnant women, the research team decided to offer the referral tool to these midwives, but to exclude them from the analyses.

After the SFP referral tool had been developed, the ten representatives of medical child and specialized child healthcare professionals (hereinafter: healthcare professionals) who were involved in the development of the referral tool were asked to bring the referral tool and the implementation study to their co-workers’ attention for recruitment and implementation purposes (e.g., by giving short presentations during meetings or sending an e-mail to their co-workers). In addition, between November 2016 and September 2018, healthcare professionals were recruited for the study via social media, the Dutch national smoking cessation website for healthcare professionals, mailings, and by presentations that the research team gave at multiple conferences and healthcare centers. All healthcare professionals that provided (medical) care to children could register online for the referral tool and the study.

 After online registration, healthcare professionals were called by the research team to receive more information on the study since the information that was provided prior to registration was limited, as this information mostly had a recruiting purpose. During this informative phone call, healthcare professionals could confirm or withdraw their registration (verbal informed consent; written informed consent was collected at the start of one of the two questionnaires**)**. In case healthcare professionals had some questions about the referral tool, these were addressed by the research team during the informative phone call. Participation in the study included healthcare professionals being asked to work with the referral tool and to complete two online short questionnaires and one semi-structured telephone interview on their experiences with the referral tool. In total, 68 healthcare professionals participated in the implementation study (see Table 2 for more information on the participant’s characteristics). After healthcare professionals had confirmed their registration for the implementation study, the research team sent the toolkit (e.g., paper-based information cards, see “Background” for more information) to them and their colleagues for free. The research team distributed 811 toolkits among the participating healthcare professionals and their colleagues (representing 54 midwifery practices^1^/hospitals/youth healthcare centers). The majority of the midwifery practices/hospitals/youth healthcare centers (*n* = 41) were represented in the study by one healthcare professional. The colleagues of these healthcare professionals did not need to participate in the study to receive a toolkit (this explains why the number of spread toolkits is substantially higher than the number of participating healthcare professionals). However, in case colleagues of participating healthcare professionals contacted the research team about the referral tool and/or implementation study, the research team invited these colleagues to participate in the study.

**Table 2:** Sample characteristics of healthcare professionals who were included in the analyses (*N* = 65)

Characteristics	*N* (%)
Female	60 (92.3 %)
Medical healthcare	52 (80.0 %)
Profession	
Pediatrician	10 (15.4 %)
Nurse	41 (63.1 %)
Specialized child healthcare professional	13 (20.0 %)
Other	1 (1.5 %)
Number of years working as healthcare professionals and seeing parents and children (*M, SD)*^a^	23.0 (10.7)

^a^Data were only available from healthcare professionals who completed the MIDI and were included in the analyses (*n* = 28)

### Data collection

During the expert meetings preliminary to the present implementation study, the representatives emphasized that it was crucial that the implementation of the SFP referral tool and the participation in the implementation study did not ask much time from the healthcare professionals. Therefore, the aims of the data collection were to prevent the overburdening of healthcare professionals on the one hand, but, on the other hand, to collect different types of data that were profound and complementary as much as possible (i.e., triangulation [29, 30]) and to strive for saturation. Because of this, healthcare professionals were invited to complete two short online questionnaires and one semi-structured telephone interview.

 The research team found it important that healthcare professionals, who were recruited after the start of the data collection, were still able to work with the SFP referral tool and to participate in the present implementation study. Because of this, the recruitment and the data collection of this study overlapped for the greater part (recruitment: from November 2016 to September 2018; data collection: from April 2017 to February 2019). An overview of the data collection is presented in Table 3. This table illustrates that the telephone interviews and two questionnaires were administered at different time points. This enabled us to collect and analyze the data concurrently. In turn, this made it possible to check and confirm the results (i.e., verification to ensure reliability and validity of the data), and look for potential gaps or omissions that could be addressed in the continuation of the study [31].

**Table 3:** Overview of the data collection for the present implementation study

	Telephone interview	First questionnaire	Second questionnaire
Timing	April 2017 – August 2017 (after HCP had worked on average 4.5 months with the SFP referral tool)	March 2018	November 2018
Instruments	Semi-structured telephone interview based on the framework of Linnan and Steckler (2002) [32]. The interview guide included seven open and closed questions	MIDI with five additional open and closed questions	Qualitative questionnaire including three open questions
Aims	To collect in-depth data on the barriers and facilitators on the implementation of the SFP referral tool (experienced by HCP)	To identify multiple determinants (e.g., self-efficacy and relevance) that affected the use of the SFP referral tool by HCP	To prioritize the barriers that HCP experienced in using the SFP referral tool and to find suggestions to overcome these barriers
Number of HCP approached^a^	35	59	62
Number of HCP completed^b^	25 (71.4 %)	31 (52.5 %)	32 (51.6 %)

Notes*.HCP* healthcare professionals; *MIDI* Measurement Instrument for Determinants of Innovations; *SFP* Smoke-free Parents

^a^ The number of approached professionals differed between the questionnaires and telephone interview, because healthcare professionals could register for the study, while the data collection had already started

^b^ Nine (13.2 %) healthcare professionals completed all three assessments

#### Semi-structured telephone interviews

Semi-structured telephone interviews were carried out so that in-depth data could be collected on the barriers and facilitators on the implementation of the SFP referral tool. After healthcare professionals had worked on average 4.5 months (range: 2.5–6 months) with the SFP referral tool, they were individually approached by telephone for a telephone interview by the primary researcher (TSvS). Because the representatives of the healthcare professionals emphasized the importance of not overburdening healthcare professionals during the data collection, several criteria determined whether healthcare professionals were approached. For example, healthcare professionals were not approached if the research team had already spoken more informally with healthcare professionals about SFP at e.g. a national conference just before the telephone interview. In addition, if healthcare professionals had already been approached for the interview and one of the two questionnaires (that were carried out at an established moment) during the same period of time, they were not approached for the telephone interview (to prevent that healthcare professionals would have been approached for both the telephone interview and a questionnaire in a short period of time). Because of these criteria, around half of the healthcare professionals (*n* = 33, 48.5 %) was not approached for the telephone interview.

For the follow-up trial, a process evaluation was conducted based on the framework of Linnan and Steckler (2002) [32]. This framework was chosen because it provides an overview on how to conduct a process evaluation for public health interventions and research. To connect with the follow-up trial and to be able to provide more insight into the barriers and facilitators that healthcare professionals experienced with the SFP referral tool, the interview guide of the telephone interview was based on several components of this framework. These components were: dose delivered, fidelity, context, dose received, and reach. The interview guide included a selection of seven closed and open questions (see appendix A). The interviews were short (about 20 min), because the research team did not want to overburden the healthcare professionals and data were also collected through the online questionnaires. All interviews were conducted in Dutch and audio recorded.

#### Online questionnaires

All healthcare professionals that participated at the time received a personal invitation by email to complete the online questionnaires. The secure web survey software application Jambo Mobile was used to collect healthcare professionals’ answers on both questionnaires. The first questionnaire that was sent to 59 healthcare professionals was the Dutch questionnaire Measurement Instrument for Determinants of Innovations (MIDI) [33]. The research team used the MIDI, because this quantitative questionnaire made it possible for the research team to obtain a broad overview of multiple determinants (e.g., self-efficacy and relevance) that affected the use of the SFP referral tool by the healthcare professionals [33]. The majority of the questions (e.g., “I have sufficient knowledge to use the referral tool”) were rated on a 5-point Likert scale ranging from (1) ‘completely disagree’ to (5) ‘completely agree’. Because researchers can select the determinants of the MIDI they are interested in, the first questionnaire included 19 closed questions and statements (covering 11 determinants of the MIDI), three questions on demographics, and two additional open and closed questions on strategies that healthcare professionals applied to motivate parents to quit smoking.

The second online questionnaire was sent to 62 healthcare professionals. Based on the data that were collected through the telephone interviews and the first questionnaire on the barriers that healthcare professionals experienced in using the SFP referral tool, the aim of the second questionnaire was to prioritize these barriers and to find suggestions to overcome these barriers. Therefore, the research team developed a short qualitative online questionnaire with three open questions in which healthcare professionals were asked to indicate the three main barriers they experienced in working with the SFP referral tool. In addition, they were asked to provide a possible solution for each mentioned barrier.

### Analyses

Of the 68 participating healthcare professionals, three were excluded from the analyses because they were a midwife or obstetrician (see for more information “Participants and recruitment”).

#### Semi-structured telephone interviews

Two research assistants transcribed verbatim the 25 audio recordings of the interviews (of which 24 were included in the analyses). Subsequently, two members of the research team (i.e., TSvS and a research assistant) inductively coded two transcripts individually (by using MAXQDA 18.2) after which the codes were discussed and the codebook was agreed on. Subsequently, based on the codebook, three more transcripts were coded individually by the same researchers and results were discussed. This process was repeated multiple times for the remainder of the transcripts whereby transcripts were individually coded and the results were subsequently discussed. Any disagreements between the two researchers were resolved by discussion, and if necessary, by consulting a third member of the research team.

After all transcripts were coded, the same researchers carried out thematic analysis, following the guidelines proposed by Braun and Clarke (2006) [34]. This means that the researchers individually searched for themes that focused on the barriers and facilitators of the SFP referral tool across all codes by looking at the relationships between the codes and grouping these codes together. Both researchers made an overview of the themes derived from the interviews, which were both discussed and improved several times until a final version was made that was discussed with the other members of the research team.

#### Online questionnaires

With respect to the first questionnaire, the closed questions were analyzed in Statistical Package for the Social Sciences, version 25. Descriptive statistics (% for categorical variables and means and standard deviations for continuous variables) are reported.

With respect to the second qualitative questionnaire, all reported barriers and suggestions to overcome the barriers were inductively coded by TSvS and discussed with RO. Thematic analysis was performed by identifying themes within and across the reported barriers and suggestions and grouping the codes together [34]. The primary researcher made an overview of the themes derived from the questionnaire, which was discussed with RO and MK. Two barriers and four suggestions that healthcare professionals reported in the second questionnaire were not categorized, because they did not fit into the themes. Although healthcare professionals were asked to number three barriers and three suggestions, not every healthcare professional did so (i.e., the questions were not mandatory). In total, missing data for each barrier or suggestion ranged from 1 to 21.

## Results

### Participant characteristics

 In total, 68 healthcare professionals participated in the present study. Table 2 presents some key characteristics of the healthcare professionals who were included in the analyses (*N* = 65).

### Facilitators, barriers, and suggestions for improvement in the implementation of the SFP referral tool

The thematic analysis of the semi-structured telephone interviews led to five overall themes in which the facilitators, barriers, and suggestions for improvement in the implementation of the SFP referral tool are integrated. From the second qualitative questionnaire, four themes were identified for the barriers (Table 4): (1) healthcare professionals experience resistance against smoking cessation among parents; (2) healthcare professionals experience lack of time to discuss smoking with parents; (3) characteristics of SFP; and (4) costs of SFP. In addition, six themes were identified for the suggestions (Table 4): (1) healthcare professionals need to align with the stages of parent’s change in quitting tobacco use; (2) some characteristics of SFP need to be changed; (3) healthcare professionals need more time to discuss smoking with parents; (4) SFP needs to be completely reimbursed; (5) healthcare professionals and parents need to receive education about smoking and how to discuss smoking; and (6) more healthcare professionals should discuss smoking cessation with parents.

**Table 4:** Main barriers and suggestions to overcome barriers in working with the SFP tool^a^

Barriers^b^	*N* = 47	Suggestions	*N* = 37
HCP experience resistance against smoking cessation among parents	23 (48.9 %)	HCP need to correspond to the stages of parent’s change in quitting tobacco use	16 (43.2 %)
Characteristics of SFP	8 (17.0 %)	Some aspects of SFP need to be changed	6 (16.2 %)
Costs of SFP	5 (10.6 %)	SFP needs to be completely reimbursed	3 (8.1 %)
Lack of time to discuss smoking with parents	6 (12.8 %)	HCP need to have more time to discuss smoking with parents	6 (16.2 %)
		More HCP should discuss smoking cessation with parents	3 (8.1 %)
		HCP and parents need to receive education about smoking and how to discuss smoking	3 (8.1 %)

Notes. *HCP* Healthcare professionals; *SFP* Smoke-free Parents

^a^ Derived from the second questionnaire

^b^ Five healthcare professionals reported they did not experience any barrier in working with the SFP tool

To provide a complete picture of the facilitators, barriers, and suggestions for improvement in the implementation of the SFP referral tool, the five overall themes derived from the telephone interviews are presented in which the results and themes from the questionnaires are integrated. The advantage of this approach is that it made it possible to compare (by confirming, strengthening or contradicting) the results that were derived from the three different data sources. Results that include percentages were derived from the questionnaires.

### Theme 1: General experiences with the SFP referral tool

Healthcare professionals reported that the tool was convenient to use and accessible. Only a few of the healthcare professionals reported that the referral tool was too difficult to use (10.7 %, *n* = 3), as noted by agreement with the question “the referral tool is too difficult to use for me” in the first questionnaire. In addition, in the first questionnaire only a few healthcare professionals reported that the referral tool did not match with the way healthcare professionals were used to discuss smoking cessation with parents (10.7 %, *n* = 3) or that the telephone counseling program SFP was not relevant for parents (10.7 %, *n* = 3). Five healthcare professionals (17.9 %) reported in the second questionnaire they did not experience any barrier in working with the SFP tool (Table 4).

Although healthcare professionals reported rather positive experiences, the majority of them mentioned in the interviews they did not use the tool often, since many parents did not want to be referred to SFP.

*“I am very happy that you started this project and it is very clear for me how to use the tool. It is just a pity that only two parents wanted to participate”* (healthcare professional 41, nurse).

Healthcare professionals mentioned that the costs, and the vagueness of the potential costs, of SFP was a major barrier for parents not being referred to SFP (see also Table 4). Related to this, one healthcare professional said that participating in SFP did not guarantee parents they would successfully quit smoking. This in combination with the potential high costs of SFP parents might have to pay made parents reluctant of being referred.

“*That makes it complicated. If you would be sure that SFP works and you quit smoking, then it is worth considering. (…) But if people do not quit and continue smoking, they pay in spades. So then they prefer spending their money on smoking over spending money on cessation assistance”* (healthcare professional 5, nurse).

More than two thirds (67.9 %, *n* = 19) of the healthcare professionals reported in the first questionnaire they were unhappy with the fact they could not provide clarity about the costs of SFP to parents.

#### Theme 2: It varies by healthcare professionals to what extent they discuss smoking with parents

Although the majority of the healthcare professionals mentioned that they regularly discussed smoking with parents, this was not the case for all healthcare professionals. For example, in the interviews most specialized youth healthcare professionals themselves indicated that they always discuss smoking cessation when they visit parents for the first time at home. However, when they see parents at a later date, specialized youth healthcare professionals indicated that they only discussed smoking cessation when there was an exceptional situation (e.g., when parents smoked a lot or when parents smoked inside). Also, healthcare professionals mentioned that they perceived large differences among their colleagues with regard to the quality and intensity with which smoking cessation was discussed with parents varied.

* “My colleagues ask about parents’ tobacco use most times. But really inventorying the reasons of parents to smoke, or assessing parent’s readiness to quit smoking, and discussing the options of cessation assistance, I think that can be improved a lot”* (healthcare professional 6, nurse).

Healthcare professionals mentioned that they did not (extensively) discuss smoking cessation with parents, because they were afraid of damaging their relationship of trust with parents.

* “It is a vulnerable topic to discuss because of the relationship of trust with parents. You need to make sure that parents do not feel pressed into the defense or being accused. (…) Yet, I can imagine that this could be a barrier for healthcare professionals to discuss this topic with parents”* (healthcare professional 7, nurse).

In addition, a couple of healthcare professionals reported that some parents were not willing to talk about smoking.

*“And with other people (…), a door closes when you want to talk about smoking with them. That is very difficult. (…) And I need to be careful, because I want them and their children to keep coming”* (healthcare professional 41, nurse).

Although healthcare professionals themselves indicated that the extent to which they discuss smoking with parents varies, the majority (89.3 %, *n* = 25) of the healthcare professionals reported in the first questionnaire that it is their job to refer smoking parents to evidence-based smoking cessation interventions.

*“I speak a bit on behalf of the child and I think it is my job to discuss it. (…) You know, sometimes I tell parents that I do not want to be accused in the future that I have never told them to quit smoking”* (healthcare professional 4, nurse).

With respect to the specialized youth healthcare professionals, this group of healthcare professionals mentioned in the interviews that it is their job to discuss smoking cessation with parents (especially when they visit parents at home for the first time). Yet, compared to medical healthcare professionals, they seem to see it less as their job to motivate parents to quit smoking and they seem to be more satisfied when parents indicate to smoke outside. Finally, the majority of healthcare professionals reported in the first questionnaire that the SFP referral tool made it easier to discuss smoking cessation (67.9 %, *n* = 19) and to help parents with quitting tobacco use (60.7 %, *n* = 17).

#### Theme 3: Healthcare professionals find it difficult to motivate parents to quit smoking and to refer them to SFP

Whereas the majority of healthcare professionals reported in the first questionnaire that they felt they were able to ask parents about their smoking status (92.8 %, *n* = 26) and to advise smoking parents to quit smoking (89.3 %, *n* = 25), fewer healthcare professionals reported they felt that they could provide motivational messages to parents to quit smoking (35.7 %, *n* = 10, self-efficacy).

*“No, I do ask the question whether they want to quit smoking. But in most cases they are not motivated to quit and then the conversation is finished”* (healthcare professional 19, pediatrician).

In the interview and the first questionnaire, healthcare professionals described several reasons that parents reported for not wanting to quit smoking. For example, parents did not experience any tobacco-related health problems, they were physically and psychologically addicted to nicotine, they were afraid they could not successfully quit smoking, they smoked outside which they thought was sufficient for their children’s health, or they experienced too much stress at the moment. Moreover, healthcare professionals mentioned that parents wanted to have some time to think about the referral before actually being referred.

*“A lot of people want to think about it first. They have not given much thought on quitting smoking, so they want to have some time for reflection”* (healthcare professional 1, nurse).

However, some healthcare professionals also thought that some parents used the reason “I want to think about it first” as an excuse for not being referred to SFP.

Although not every healthcare professional mentioned to be in need of training on how to discuss smoking cessation with parents and how to motivate them to quit smoking, some healthcare professionals pointed out they wanted to receive training. Further, healthcare professionals mentioned that, in addition to the materials of the SFP toolkit, they wanted to receive some practical and easy accessible tools (e.g., example sentences and informative and motivational materials they could give to parents) they could use to motivate parents to quit smoking.

#### Theme 4: Healthcare professionals are not the parent’s, but the child’s healthcare provider

One of the characteristics of the SFP referral tool is that healthcare professionals can offer evidence-based smoking cessation treatment to parents via their children. However, multiple healthcare professionals mentioned they experienced tension when they tried to motivate parents to quit smoking and to refer them to SFP, since they were not the parent’s, but the child’s healthcare provider.

* “Look, the difficulty is that parents are not our patients. If parents would be my patients, I would feel more freedom to make a telephone appointment at a later moment to discuss their smoking. But when you make a follow-up appointment with parents while they are not your patients, you are treating them”* (healthcare professional 1, nurse).

Related to this, multiple healthcare professionals said that some parents did not expect healthcare professionals to discuss their smoking and to refer them to SFP during a consult that was marked by their children’s, and not their own, health.

*“Yesterday I had a mother of a patient who thought she came to see me for her child. She was completely overwhelmed by me. She said she was here for her child, and now we were talking about her. So I replied that I understood her feelings, but her behavior was related to her child’s health”* (healthcare professional 7, nurse).

#### Theme 5: Healthcare professionals have limited time to discuss smoking cessation with parents

The majority of healthcare professionals mentioned that an important barrier for not extensively discussing smoking cessation with parents was that they had limited time to do so (Table 4). Some healthcare professionals reported that their consults with parents and children lasted between ten and 15 min in which multiple topics needed to be discussed. In addition, when children experienced health problems that were not related to their parent’s smoking, it was seen as less relevant to discuss smoking with parents when there was not a lot of time.

*“There are quite a few patients who visit us because their children have health problems that are not related to their parent’s smoking. Even though smoking is bad for them, parents and their children come for something else. In these cases, the focus of the consult is not on the parent’s smoking”* (healthcare professional 19, pediatrician).

Also, multiple healthcare professionals reported that parents and their children visited them only a few times a year, which delays the process in helping parents to quit smoking. Healthcare professionals reported that they needed more time to discuss smoking cessation with parents as a suggestion for this major barrier.

## Discussion

The present implementation study examined the facilitators, barriers, and suggestions for improvement in the implementation of the proactive SFP referral tool in child healthcare settings. Both groups of child healthcare professionals (i.e., medical child healthcare professionals and specialized child healthcare professionals) found the tool accessible and convenient to use. Yet, they also reported that several barriers limited their use, and thus the implementation, of the tool.

The first barrier that made the implementation of the SFP referral tool difficult is that healthcare professionals faced resistance to smoking cessation (or even talking about smoking cessation) among parents and, as a result, found it difficult to refer parents to SFP. According to healthcare professionals, parents had various reasons for not wanting to quit smoking or being referred to SFP (e.g., they experienced too much stress). These reports by Dutch healthcare professionals are not completely in line with previous studies from the United States showing that most parents were positive about talking about smoking with their child’s healthcare professional and that the majority of parents was open to receive some help to quit smoking (e.g., receiving information about where to get assistance) [35–37]. More specifically, several American studies found high percentages of parents accepting (or reporting to be willing to accept) to be referred to a quitline if offered by a child’s healthcare professional. For example, a study found that 60 % of the 187 smoking parents would accept enrollment in a telephone smoking cessation counseling intervention if offered by a child’s doctor [38]. Although we do not know the exact proportion of parents that agreed to be referred to SFP, based on the interviews with the healthcare professionals it is likely lower than 60%.

A possible explanation for these differences in parent’s attitudes and acceptance rates could be cultural. In Dutch culture, personal choice and compromise and negotiation (instead of conflict) are highly valued [39]. Because of this, Dutch healthcare professionals might feel less comfortable in addressing smoking cessation and prefer to find a compromise (e.g., try to motivate parents to smoke outside the house). It may also be that the perceptions of healthcare professionals are primarily based on a couple of negative responses of parents and not on the majority positive responses of parents (the negativity bias) [36, 40, 41]. Further research into parent attitudes toward smoking cessation assistance by child healthcare professionals is needed, as, to the best of our knowledge, there has been no nationwide study that examined parent’s attitudes towards addressing parental smoking in child healthcare settings in the Netherlands. Assessing these attitudes could provide insight into whether the perceptions of healthcare professionals on parent’s attitudes towards addressing smoking correspond to the actual perceptions of parents themselves. A qualitative study could be performed to explore the parent’s attitudes after which a larger quantitative study could be carried out to examine which attitudes are most prevalent among parents and whether any subgroups of parents with different attitudes exist.

Another explanation for the resistance that healthcare professionals faced to smoking cessation among parents could be the less optimal timing of addressing the parent’s smoking behavior. Our data show that multiple healthcare professionals reported that some parents had not foreseen that their smoking would be addressed during a visit with their child’s healthcare provider. Further, the results from the interviews illustrate that some healthcare professionals experienced tension when motivating parents to quit smoking, as they were not the parent’s, but the child’s healthcare provider. Both aspects could lead to situations in which parents and healthcare professionals feel less comfortable to discuss smoking cessation, and thus limits the implementation of the SFP referral tool in child healthcare settings.

A final major barrier that made the implementation of the SFP referral tool difficult concerns the potential costs of SFP that parents had to pay. In the Netherlands it is obligatory for people to have health insurance [42]. At the time that the present study was carried out, health insurance agencies reimbursed evidence-based smoking cessation interventions once a year depending on the health insurance that smokers had and whether smokers already used their deductible (i.e., depending on the type of care that people need, they first need to pay their annual deductible). Healthcare professionals reported that the high costs of SFP (between € 300,00 and € 400,00) and the vagueness of whether parents needed to pay for SFP, caused that parents did not want to be referred to SFP. This is a missed opportunity for the implementation of SFP in the Netherlands. A Cochrane review showed that full reimbursement of smoking cessation interventions increased the use of smoking cessation behavioral interventions and the abstinence rates at six months or longer [43]. From 2020, people’s deductible is, under certain circumstances, not applicable on evidence-based smoking cessation treatment anymore in the Netherlands [44]. This means that it has become less likely that parents have to pay for their treatment. Yet, despite this recent positive development, the implementation of the SFP referral tool in child healthcare settings and the acceptance rate of SFP among parents can substantially be improved if health insurance agencies fully reimburse SFP for parents [43].

### Implications for practice and directions for future research

The present study provides two main implications to improve the implementation of the SFP referral tool in child healthcare settings. First, in the current approach of the SFP referral tool healthcare professionals need to motivate parents to quit smoking before they can refer parents to SFP. However, the present study (and previous published studies) found multiple barriers that limited the use SFP referral tool among healthcare professionals. For example, healthcare professionals experience a lack of time to discuss smoking [45–50], healthcare professionals see parents only a few times a year [49], healthcare professionals are afraid to damage their relationship of trust with parents [49], healthcare professionals feel less skilled to motivate parents to quit smoking [47–49], healthcare professionals find it less relevant to discuss smoking with parents when children do not experience any smoking-related health problems [49], and healthcare professionals are not the parent’s, but the child’s healthcare provider. Perhaps a better approach could be if healthcare professionals only assess parent’s smoking status and ask smoking parents whether they would be open to be proactively contacted by another professional (e.g., clinical social workers or counselors) to talk about their smoking (and other health-related lifestyle topics if necessary) more extensively. During this informal conversation, this professional could correspond to the stages of parent’s change [51] in quitting tobacco use and apply various techniques, including motivational interviewing, problem-solving, and cognitive behavioral skill building [52]. In case parents are motivated to quit smoking, the professionals could ask whether parents are willing to be referred to SFP. Perhaps, parents react more positively to the question whether they are willing to be referred to another professional to discuss their smoking in general than to the question whether parents want to be referred to SFP, since parents do not already need to be willing to quit smoking. Future research could explore whether this new approach could be effective in overcoming these barriers and could improve the implementation of the SFP referral tool in child healthcare settings.

A second approach to improve the implementation of the SFP referral tool concerns the costs of SFP. In case health insurance agencies are not able to fully reimburse the costs of SFP for parents, it is important that healthcare professionals are better able to inform parents about the potential costs. Therefore, a potential interim solution could be to develop an easy accessible online tool that healthcare professionals could use to inform parents about the potential costs of SFP. More specifically, by answering some questions about the health insurance of parents (e.g., what health insurance do parents have) in an online tool, healthcare professionals are better able to make an estimation about the potential costs of SFP and inform parents.

#### Limitations

The present study had several limitations. First, the healthcare professionals who participated in this study were limited to only a few different types of healthcare professionals (e.g., pediatricians and pediatric nurse practitioners). In the Netherlands, other types of healthcare professionals (e.g., maternity nurses) also work in pediatric settings (in primary care) and are potential users of the SFP referral tool. Currently, it is unknown whether the results of the present study are generalizable to these other types of healthcare professionals. To enhance broad implementation of the SFP tool among healthcare professionals in child healthcare settings, future research could explore whether the SFP referral tool could also be used by other types of healthcare professionals and whether any (small) adaptations to the tool are needed (tailoring). Second, the fact that the colleagues of the participating healthcare professionals were not asked to participate in the present study limits the representativeness of the results of this study (e.g., 92.3 % of the participants was female). The reason that we decided not to ask the colleagues of the participating healthcare professionals was because the representatives of the professionals strongly advised us to not overburden the healthcare professionals. Related to this, to make the questionnaires as short as possible we only asked questions about the type of profession in the first questionnaire and not in the second questionnaire. Because of this, we were unable to identify any patterns in terms of type of professional among healthcare professionals who reported no barriers in the second questionnaire. Fourth, for implementation purposes, the recruitment of healthcare professionals and the data collection among healthcare professionals partially overlapped. After the first round of interviews and questionnaires was conducted, new healthcare professionals were still joining the study. Therefore, not all healthcare professionals were approached for both questionnaires and the telephone interview which resulted in less collected data. However, we expect the consequences of the different distribution of healthcare professionals over the interview and questionnaire rounds to be minimum, because saturation of information seemed to be reached after the last interviews and round of questionnaires. Finally, due to the study design we were unable to assess how many parents could have been referred to SFP (i.e., how many parents that were seen by healthcare professionals smoked and wanted to quit smoking).

## Conclusions

The present implementation study showed that healthcare professionals found the SFP referral tool easy accessible and convenient to use. However, the use of the tool among healthcare professionals was limited due to several barriers that they experienced in the implementation. The main barriers concerned the resistance that healthcare professionals faced to smoking cessation among parents and the potential high costs of SFP. To increase the impact of the evidence-based telephone smoking cessation counseling SFP via child healthcare settings, it is important to overcome these barriers. Future research could examine whether more parents will be referred to SFP after they have been referred to a clinical counselor to discuss their smoking. In addition, health insurance agencies need to be encouraged to fully reimburse SFP. These changes could improve the implementation of the SFP referral tool in child healthcare settings and result in potentially more parents quitting smoking.

## Data Availability

The datasets used and/or analysed during the current study are available from the corresponding author on reasonable request.

## Appendix A: Interview guide for the semi-structured telephone interviews

The following topics were discussed during the semi-structured telephone interviews (not specifically in the order below):

1. Did the healthcare professionals receive the Smoke-free Parents (SFP) toolkit and did they distribute the toolkit among their colleagues (dose delivered)?

2. Did the healthcare professionals use the SFP referral tool as supposed to (fidelity)?

3. Satisfaction of healthcare professionals with the SFP referral tool (dose received)

4. Contextual factors that affect the implementation of the SFP referral tool (context)

5. Frequency of referrals to SFP by healthcare professionals (reach)

6. What is the reason that so few parents want to be referred to SFP and how can this number be increased? (reach)

7. To what extent do healthcare professionals use the SFP referral tool when they see parents who smoke? And to what extent do they discuss smoking (cessation) with parents? (dose received/reach)

## Abbreviations

AAR: Ask-advise-refer
HCP: Healthcare professionals
MIDI: Measurement Instrument for Determinants of Innovations
RCT: Randomized controlled trial
SFP: Smoke-free Parents
SHS: Secondhand smoke

## Footnotes

1 Healthcare professionals providing antenatal care were not the target group of the SFP referral tool. However, an exception was made for three midwifery practices that indicated to be in need of the SFP referral tool (see for more information “Participants and recruitment”).
